# Polymerization Kinetics of Conventional vs. Fiber-Reinforced Composites Using Rapid High-Intensity Photo-Activation

**DOI:** 10.3390/polym18161931

**Published:** 2026-08-07

**Authors:** Theresa M. Fischer, Matej Par, Daniel Miler, Tobias T. Tauböck

**Affiliations:** 1 Department of Conservative and Preventive Dentistry, Center for Dental Medicine, University of Zurich, 8032 Zurich, Switzerland; tobias.tauboeck@zzm.uzh.ch; 2 Department of Endodontics and Restorative Dentistry, University of Zagreb School of Dental Medicine, 10000 Zagreb, Croatia; mpar@sfzg.unizg.hr; 3 University of Zagreb, Faculty of Mechanical Engineering and Naval Architecture, 10002 Zagreb, Croatia; daniel.miler@fsb.unizg.hr

**Keywords:** degree of conversion, short-fiber-reinforced composites, high-intensity photo-polymerization, ATR-FTIR spectroscopy

## Abstract

This in vitro study examined the effects of rapid high-intensity vs. conventional light-curing on real-time degree of conversion (DC) of conventional and fiber-reinforced resin composites. Two resin composites specifically designed for rapid high-intensity photo-polymerization (Tetric PowerFill, Tetric PowerFlow), two flowable fiber-reinforced composites (everX Flow, FibraFill Flow), and two condensable fiber-reinforced composites (everX Posterior, FibraFill DENTIN) were included. Photo-activation was performed using two protocols: 3 s at 3006 mW/cm^2^ or 10 s at 1108 mW/cm^2^. DC of 1.5 mm thick specimens (*n* = 6) was monitored for 5 min by real-time ATR-FTIR spectroscopy, and the maximum polymerization rate (R_DCmax_) was determined. Data were analyzed using independent *t*-tests and ANOVA followed by Tukey’s post hoc test (α = 0.05). DC at the end of the 5 min observation period ranged from 33.7 ± 0.9% (FibraFill DENTIN, 3 s) to 59.2 ± 0.3% (Tetric PowerFlow, 10 s). All composites exhibited a significantly higher DC with the 10 s curing protocol compared with 3 s high-intensity light-curing. Within both curing protocols, Tetric PowerFlow, everX Flow, and everX Posterior achieved the highest DC values, whereas FibraFill DENTIN showed the significantly lowest DC. R_DCmax_ was significantly higher with the 3 s protocol than with 10 s light-curing for all materials, with the significantly highest values recorded for Tetric PowerFill and Tetric PowerFlow. FibraFill DENTIN and FibraFill Flow exhibited the significantly lowest R_DCmax_ for both curing protocols. In conclusion, 3 s high-intensity photo-activation increased the polymerization rate but simultaneously reduced the degree of conversion of conventional and fiber-reinforced flowable and condensable resin composites compared with a conventional 10 s light-curing regimen.

## 1. Introduction

In modern restorative dentistry, the use of conventional resin-based composites has become the standard approach for direct restoration of teeth affected by caries or structural defects [1,2]. In recent years, contemporary restorative dentistry has undergone significant advancements driven by the development of materials and techniques aimed at streamlining clinical procedures and enhancing the interaction between restorative materials and dental hard tissues. Among these innovations, bulk-fill resin composites have gained particular attention, as they allow for placement in increments of up to 4–5 mm. This approach reduces the complexity and duration of restorative procedures and may decrease technique sensitivity, while improving physical properties and reducing polymerization stress [3,4,5,6,7,8,9]. A study has demonstrated that 4 mm bulk-fill resin composites exhibit marginal integrity that is at least similar, or even superior, to that of conventional composites applied using the standard 2 mm layering technique [10].

Moreover, restorative workflows can be further optimized by high-intensity light-curing units, which enable polymerization within very short exposure times of only 3 s and irradiance levels above 3000 mW/cm^2^ [11,12,13]. Such approaches could further reduce chairside time while aiming to achieve adequate polymerization of the composite material [14]. Novel photo-polymerization strategies have been introduced, including the incorporation of β-allyl sulfone-based addition–fragmentation chain transfer (AFCT) agents into the resin matrix of specifically designed bulk-fill composites [15,16]. This modification has been shown to improve network rearrangement during polymerization, thereby increasing thermal stability and allowing for ultrafast light-curing protocols without adversely affecting mechanical performance or the degree of conversion (DC) [15].

Reversible addition–fragmentation chain transfer (RAFT) polymerization has also been introduced into dentistry. Originally developed in polymer science, this approach employs thiocarbonylthio-based chain transfer agents (RAFT agents) to reversibly regulate propagating radicals during polymerization [15,17,18]. Although both AFCT and RAFT rely on addition–fragmentation reactions, RAFT further establishes a reversible equilibrium between active and dormant polymer chains, thereby providing greater control over polymerization kinetics. During this process, the propagating radical temporarily reacts with the RAFT agent to form a transient intermediate, which subsequently fragments to regenerate a new active radical while the growing polymer chain enters a dormant state [18]. This dynamic equilibrium delays irreversible chain termination, promotes the formation of a more homogeneous polymer network, and has been associated with improved DC and reduced polymerization shrinkage stress [19,20].

However, beyond light-curing protocols and photochemical modifications, the intrinsic composition of the composite material itself plays a critical role in determining polymerization behavior and mechanical performance. A growing body of in vitro evidence, supported by findings from systematic reviews, indicates that the incorporation of fiber reinforcement into resin matrix systems, particularly in the form of short-fiber-reinforced composites (SFRCs), can significantly improve the mechanical stability of restored teeth [21,22,23]. Short, randomly distributed glass fibers incorporated into composite materials serve as internal reinforcement elements that hinder crack initiation and propagation, thereby redistributing stresses within the restoration [24]. In contrast, long or continuous polyethylene fibers, such as those used in Ribbond systems, mainly provide a splinting effect by stabilizing cuspal structures and facilitating the redistribution of tensile and shear forces along axial walls [25,26].

Compared to conventional composite materials, SFRC materials have been shown to improve fracture resistance when used as base materials in high-stress-bearing restorations [27]. Moreover, fractures occurring in conjunction with SFRC are more frequently characterized by less catastrophic patterns above the cemento-enamel junction, which are considered more favorable to clinical repair and may therefore contribute to improved long-term treatment outcomes [28,29,30,31]. Given their promising mechanical properties and behavior, SFRCs are expected to become increasingly relevant in clinical restorative dentistry.

Despite these documented advantages, limited information is available regarding the polymerization behavior of SFRC materials, particularly under contemporary high-intensity light-curing protocols. The DC represents a key parameter for quantifying the extent of polymerization in methacrylate-based resin composites, reflecting the proportion of C=C double bonds converted into single bonds [32]. It is closely associated with both mechanical performance and biocompatibility, as higher conversion levels result in improved material properties and reduced residual monomer content [32]. Adequate polymerization is essential, as insufficient curing may lead to marginal defects, leakage, and potential pulpal complications [33,34], ultimately compromising restoration longevity [35]. Although numerous studies have investigated the mechanical properties of SFRCs [36,37,38,39], evidence regarding their polymerization behavior and kinetics remains scarce [40,41]. While rapid high-intensity photo-polymerization has been investigated for conventional bulk-fill resin composites [42,43], its influence on the polymerization behavior of SFRC has not yet been evaluated. Due to their distinct composition and fiber-reinforced microstructure, the response of SFRCs to rapid high-intensity curing cannot be assumed to be comparable to that of conventional composites. Given that both the applied light-curing protocol and material composition may affect polymerization kinetics and DC, a deeper understanding of these interactions is essential.

Therefore, the present study aims to evaluate the influence of high-intensity vs. conventional photo-activation protocols on DC and polymerization kinetics of conventional and short-fiber-reinforced composites. The null hypotheses tested were that (1) the light-curing protocol would not affect polymerization kinetics and DC, and that (2) different types of composite materials would not exhibit different DC and polymerization kinetics.

## 2. Materials and Methods

### 2.1. Composite Materials, Light-Curing Protocols, and Specimen Preparation

Six resin composites were examined, including both conventional and short-fiber-reinforced composite materials: two conventional bulk-fill composites (Tetric PowerFill and Tetric PowerFlow, both from Ivoclar, Schaan, Liechtenstein), two flowable short-fiber-reinforced composites (everX Flow, GC Corporation, Tokyo, Japan, and FibraFill Flow, ADM, Brno, Czech Republic), and two condensable short-fiber-reinforced composites (everX Posterior, GC Corporation, Tokyo, Japan, and FibraFill DENTIN, ADM, Brno, Czech Republic). Among these materials, Tetric PowerFill and Tetric PowerFlow were specifically developed for high-intensity light-curing. The manufacturers’ information, classification, and composition of the composite materials used in the present study are depicted in Table 1.

The samples of resin composites were photo-activated using a violet–blue LED curing device (Bluephase PowerCure, Ivoclar, Schaan, Liechtenstein; emission wavelength range 390–500 nm) following the two light-curing protocols: a “3 s” protocol, applying high-intensity light at 3006 mW/cm^2^ for 3 s (radiant exposure: 9.0 J/cm^2^), and a “conventional” protocol, using an intensity of 1108 mW/cm^2^ for 10 s (radiant exposure: 11.1 J/cm^2^). The radiant exitance was measured and routinely verified using a calibrated UV-Vis spectrophotometer system with NIST reference standards (MARC, BlueLight Analytics, Halifax, NS, Canada).

### 2.2. Degree of Conversion

Real-time degree of conversion was measured using an attenuated total reflectance Fourier-transform infrared spectrometer (ATR-FTIR; Nicolet iS50, Thermo Fisher, Madison, WI, USA). For this purpose, composite specimens were produced in cylindrical silicone molds measuring 3 mm in diameter and 1.5 mm in thickness, after which they were carefully positioned on a diamond ATR crystal. The bottom surface of each specimen was maintained in direct contact with the crystal, whereas the upper surface was covered with a polyethylene terephthalate (PET) film. Photo-activation was performed by placing the light-curing unit directly above the specimen surface. Infrared spectra were acquired continuously for 5 min after photo-activation, at 2 spectra per second, with four co-added scans per spectrum and a spectral resolution of 8 cm^−1^. For each experimental group, six independent replicates were performed.

Real-time DC was computed from the ratio of the aliphatic C=C absorbance at 1638 cm^−1^ to the absorbance of the aromatic C–C reference band at 1608 cm^−1^ [44]. DC as a function of time (%) was calculated as

$$DC(\%)=\left[1-\frac{{\left(\;A_{1638\;{cm}^{-1}}/A_{1608\;{cm}^{-1}}\right)}_{t\;}\;}{{\left(\;A_{1638\;{cm}^{-1}}/A_{1608\;{cm}^{-1}}\right)}_{t=0})}\right]\times 100$$

The final DC measured at the end of the 5 min observation period was defined as DC_5min_. To assess polymerization kinetics, DC vs. time curves were differentiated with respect to time (dDC/dt) to obtain the polymerization rate [43]. The polymerization rate vs. time profile was then used to identify the maximum reaction rate (R_DCmax_) [43].

### 2.3. Statistical Analysis

Normality was assessed using normal Q-Q plots and the Shapiro–Wilk test, while homogeneity of variances was evaluated using Levene’s test. As the assumptions for parametric statistics were met, DC_5min_ and R_DCmax_ were analyzed using a two-way analysis of variance (ANOVA) with the factors “material” and “curing protocol”. Since significant interactions between the factors were observed, simple main effects were analyzed using one-way ANOVA for comparisons among materials and independent-samples *t*-tests for comparisons between curing protocols. Multiple comparisons among materials were adjusted using Tukey’s post hoc test. The significance level was set at α = 0.05. Statistical analyses were performed using SPSS Statistics, version 25.0 (IBM, Armonk, NY, USA).

## 3. Results

**Figure 1:**
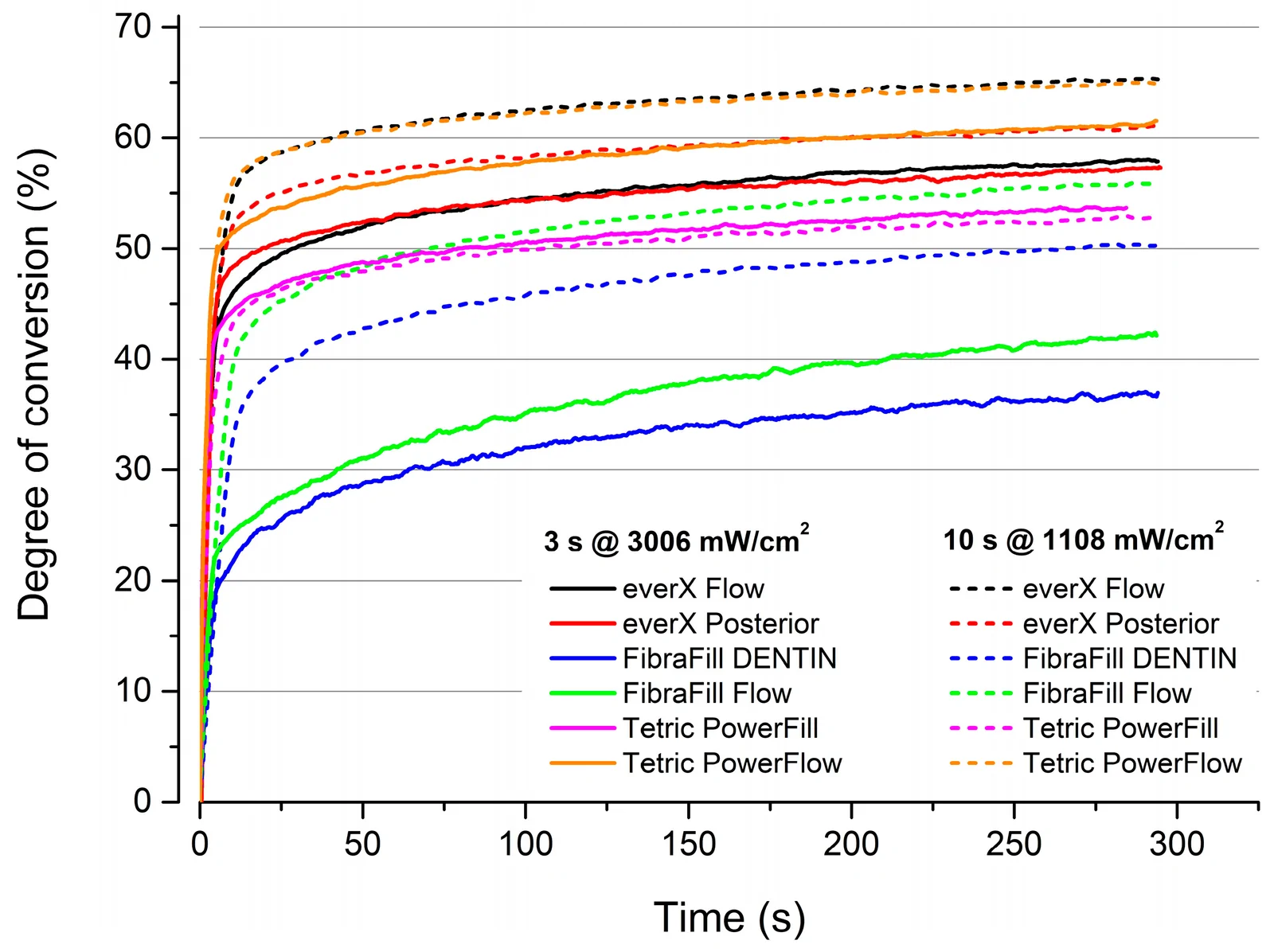
Real-time degree of conversion as a function of time for the investigated resin composites photo-activated using either a 3 s high-intensity protocol or a conventional 10 s protocol.

**Figure 2:**
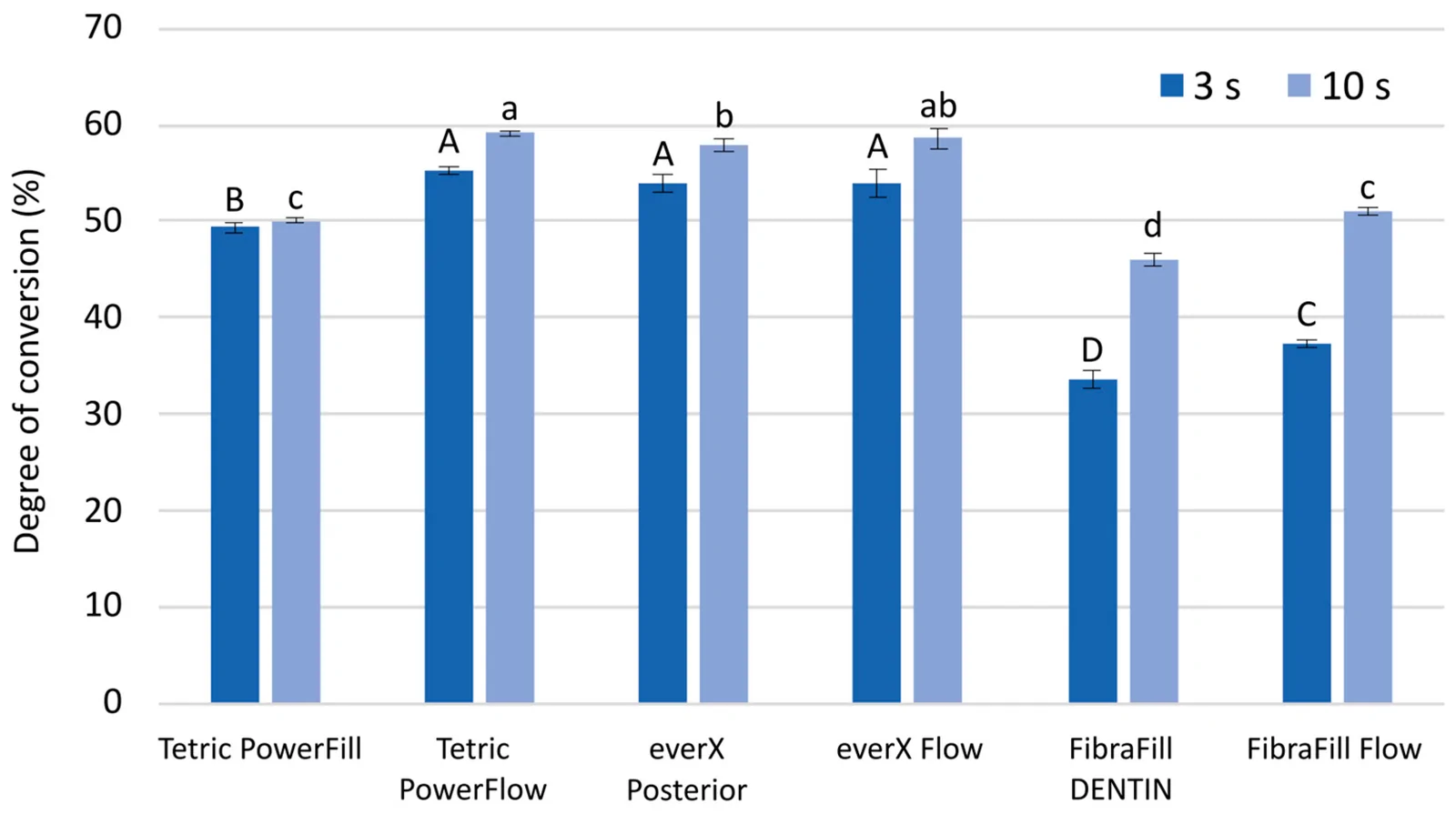
Degree of conversion measured 5 min after photo-activation (mean values ± standard deviation). The same uppercase letters denote statistically homogeneous groups for the 3 s curing protocol, whereas the same lowercase letters denote statistically homogeneous groups for the 10 s curing protocol.

**Figure 3:**
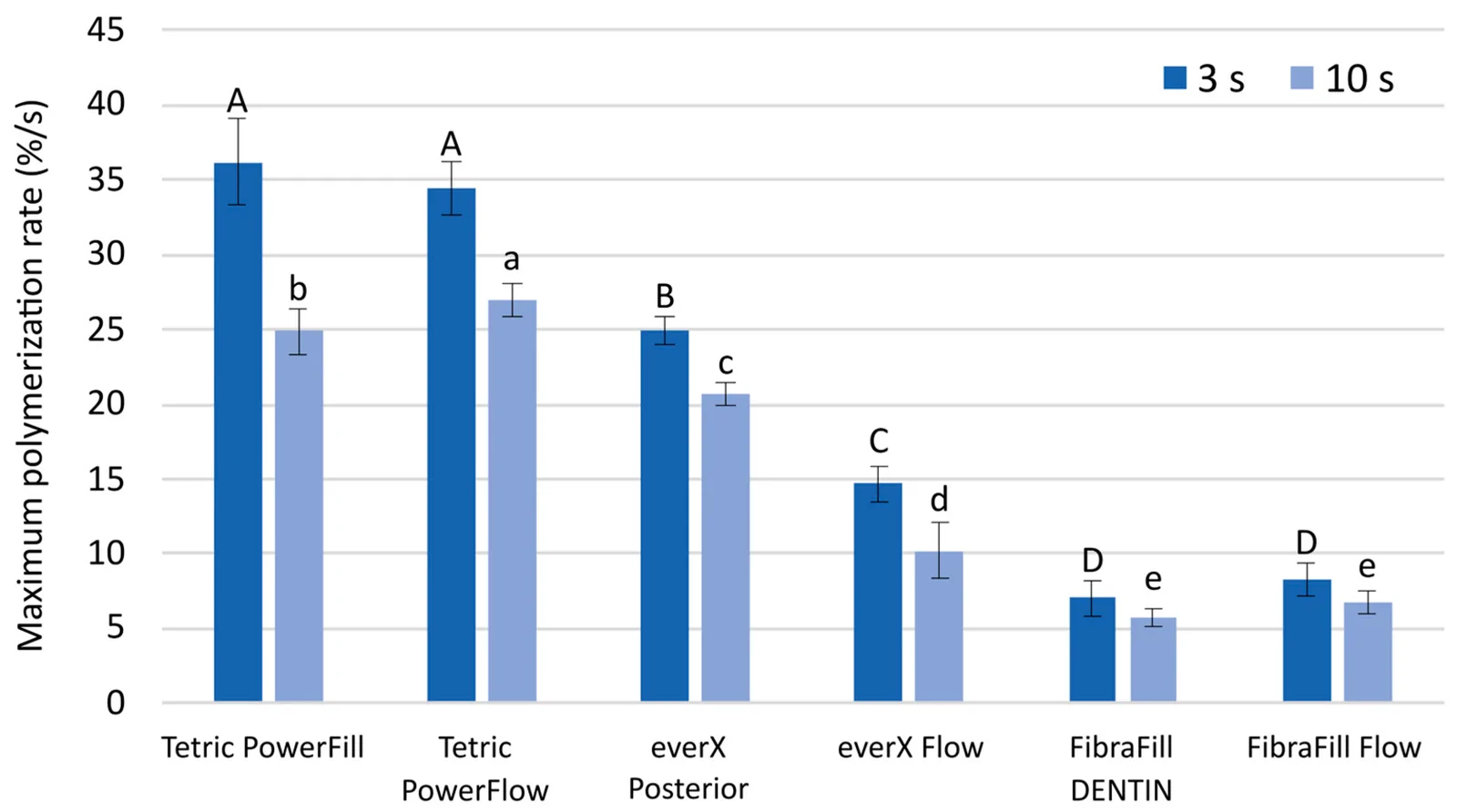
Maximum polymerization rate (mean values ± standard deviation). The same uppercase letters denote statistically homogeneous groups for the 3 s curing protocol, whereas the same lowercase letters denote statistically homogeneous groups for the 10 s curing protocol.

Figure 1 shows the real-time development of DC of the conventional and short-fiber-reinforced resin composites under high-intensity and conventional photo-activation. Figure 2 displays the DC measured 5 min after the start of polymerization (DC_5min_), and Figure 3 the maximum polymerization rate (R_DCmax_). For both DC_5min_ and R_DCmax_, two-way ANOVA identified significant effects of the factors “material” (DC_5min_: *p* < 0.001, partial η^2^ = 0.989; R_DCmax_: *p* < 0.001, partial η^2^ = 0.984) and “curing protocol” (DC_5min_: *p* < 0.001, partial η^2^ = 0.958; R_DCmax_: *p* < 0.001, partial η^2^ = 0.791), as well as significant “material” × “protocol” interactions (DC_5min_: *p* < 0.001, partial η^2^ = 0.923; R_DCmax_: *p* < 0.001, partial η^2^ = 0.645). The significant material × curing protocol interactions indicate that the influence of the curing protocol depended on the composite material, with some materials showing greater differences in DC_5min_ and R_DCmax_ between the two curing protocols than others. Thus, simple main effects were analyzed using independent-samples *t*-tests and one-way ANOVAs to determine the effect of the curing protocol within each material and the effect of the material within each curing protocol, respectively. DC_5min_ ranged from 33.7 ± 0.9% (FibraFill DENTIN, 3 s) to 59.2 ± 0.3% (Tetric PowerFlow, 10 s). Across all tested composites, 10 s conventional light-curing resulted in significantly higher DC values compared to 3 s high-intensity photo-activation. Tetric PowerFlow, everX Flow and everX Posterior exhibited the highest DC within the 3 s light-curing protocol, with Tetric PowerFlow and everX Flow also showing the highest DC values under conventional 10 s light-curing (Figure 2). In contrast, FibraFill DENTIN showed the significantly lowest DC for both curing protocols. FibraFill Flow exhibited the lowest DC among the flowable materials, irrespective of the curing protocol.

The maximum polymerization rate (R_DCmax_) ranged from 5.8 ± 0.6%/s (FibraFill DENTIN, 10 s) to 36.2 ± 2.8%/s (Tetric PowerFill, 3 s) and was significantly higher at 3 s vs. 10 s light-curing for all composite materials. With both light-curing protocols, the highest R_DCmax_ were recorded for Tetric PowerFill and Tetric PowerFlow, whereas FibraFill DENTIN and FibraFill Flow exhibited the significantly lowest R_DCmax_, irrespective of the curing protocol.

## 4. Discussion

The present study demonstrated that the applied light-curing protocol significantly affected the DC of all investigated composite materials. Across all composites, the 10 s light-curing protocol resulted in significantly higher DC values compared to 3 s high-intensity photo-activation. This finding can primarily be attributed to the higher total radiant energy of 11.1 J/cm^2^ delivered during the 10 s protocol, which likely promoted a more extensive polymerization reaction in the materials. Although high-intensity 3 s curing offers the clinical advantage of reduced chairside time, the lower total energy delivered may limit conversion and thereby compromise the final DC, which may further be exacerbated by greater susceptibility to bimolecular termination [45]. From a clinical perspective, these findings indicate that replacing conventional light-curing with rapid high-intensity photo-activation may not be universally applicable to all composite materials. While ultrafast curing protocols can substantially improve clinical efficiency, their successful implementation appears to depend on the material formulation and its ability to maintain an adequate DC under high irradiance. Previous studies have shown that a higher DC is associated with improved physico-mechanical properties of resin composites [46,47], further emphasizing the clinical relevance of the present findings.

The minor differences in DC between the two light-curing protocols for Tetric PowerFill (1.4%) and Tetric PowerFlow (6.6%) are the smallest among all investigated composites and may be explained by modified resin chemistry, which incorporates addition–fragmentation chain transfer (AFCT) technology based on β-allyl sulfone groups [48]. It is often discussed in relation to reversible addition–fragmentation chain transfer (RAFT) mechanisms [15], since the primary function of AFCT in restorative composites is the promotion of network rearrangement during polymerization [48]. Through reversible bond exchange reactions, stress concentrations developing during rapid polymerization can be relieved, allowing a more homogeneous polymer network to form. This mechanism improves reactive species mobility even at high conversion levels and helps maintain efficient polymerization under ultrafast high-intensity curing conditions. As a result, materials such as Tetric PowerFill and Tetric PowerFlow may achieve a similar polymerization efficiency with high-intensity and conventional light-curing protocols, while simultaneously reducing polymerization shrinkage stress and preserving mechanical properties [49]. The favorable performance of Tetric PowerFill and Tetric PowerFlow under rapid high-intensity curing may also be attributed to their advanced photoinitiator system. In addition to camphorquinone, these materials contain the benzoyl germanium photoinitiator Ivocerin [50], a Norrish type-I photoinitiator that generates at least two initiating free radicals per activated molecule, thereby increasing polymerization efficiency compared with conventional camphorquinone-based systems [50]. When combined with a light-curing unit specifically designed to match its absorption spectrum, this photoinitiator system is particularly well suited for ultrashort light-curing protocols. Similar findings have been reported for experimental composites containing type-I photoinitiators, where materials specifically optimized together with matching light-curing units exhibited higher DC and improved mechanical properties under shorter light-curing times than conventionally cured camphorquinone-based composites [51].

In contrast, the SFRCs FibraFill DENTIN and FibraFill Flow exhibited the highest percentage differences between the 10 s and 3 s light-curing protocol, both approaching 27%, followed by everX Flow and everX Posterior with differences of 8.1% and 6.8%, respectively. A possible explanation for the more pronounced differences observed in the FibraFill materials may be related to their material design, as these composites are not specifically engineered for ultrafast high-intensity light-curing protocols. In addition, differences in translucency and optical behavior may have contributed to the observed results. Compared with everX Posterior, which is approved for increment thicknesses up to 4 mm, FibraFill materials may exhibit reduced light transmission and increased light scattering, thereby rendering polymerization more dependent on irradiance and consequently more sensitive to variations in the applied curing protocol.

A similar influence of curing protocols was observed for the maximum polymerization rate. Across all investigated composites, 3 s high-intensity photo-activation resulted in significantly higher R_DCmax_ compared to the 10 s protocol. This can be attributed to the substantially higher irradiance applied over a shorter period, which promotes rapid radical generation and leads to a steeper initial polymerization reaction [52]. However, this accelerated reaction is typically accompanied by earlier network vitrification, which may limit further conversion despite the high initial reaction rate [53]. Tetric PowerFill and Tetric PowerFlow exhibited the significantly highest R_DCmax_ values for both curing protocols, as well as the largest relative differences in R_DCmax_ between the 3 s and 10 s conditions. This behavior is likely related to their modified resin systems incorporating AFCT chemistry. While the high R_DCmax_ primarily results from the elevated irradiance, the AFCT mechanism enables network rearrangement during polymerization, delaying vitrification and maintaining molecular mobility for a longer period. This may partially compensate for the rapid polymerization reaction induced by high-intensity curing, allowing these materials to better sustain conversion, despite the accelerated kinetics, resulting in high DC.

With the exception of everX Flow and everX Posterior, the flowable composites generally exhibited significantly higher DC compared to their higher filled condensable counterparts. Higher filler loading reduces the volume fraction of the polymerizable resin matrix and may restrict the mobility of propagating radicals, thereby limiting the extent of polymerization [54]. Consequently, the increased matrix content of flowable composites provides a greater fraction of polymerizable monomers and enhances molecular mobility during polymerization, facilitating a more efficient polymerization [55]. In addition, the lower viscosity of flowable materials [56] allows for improved radical diffusion and delayed vitrification, which may further contribute to a higher final DC level [57,58]. However, under high-intensity curing conditions, these materials may also be more susceptible to bimolecular termination and premature loss of free radicals, potentially counteracting these advantages [59,60].

Interestingly, everX Posterior and everX Flow achieved DC values similar to those of Tetric PowerFlow and even higher than those of Tetric PowerFill for both light-curing protocols, indicating a favorable curing performance of these SFRC materials. Aligning with our findings, in a previous study, everX Flow also exhibited the highest DC when compared with conventional resin-based composite materials [61]. In contrast, FibraFill DENTIN and FibraFill Flow showed the lowest DC values with both light-curing protocols. These differences may be attributed to variations in both fiber architecture and resin matrix composition. While short glass fibers can restrict monomer mobility and alter light transmission due to scattering effects, thereby limiting polymerization efficiency [62], the presence of triethylene glycol dimethacrylate (TEGDMA) in the everX materials was previously proven to enhance DC due to its low viscosity [63]. The low viscosity of TEGDMA increases molecular mobility [64,65] and facilitates radical diffusion and may additionally exert a synergistic effect on the polymerization rate, allowing the reaction to proceed further before network rigidification occurs [66]. Notably, FibraFill Flow exhibited the significantly lowest DC among all flowable materials despite its relatively low filler loading (~60 wt%) and the presence of TEGDMA, suggesting that fiber-related effects may outweigh the beneficial influence of matrix composition in this material.

The investigated everX and FibraFill SFRC materials differ in fiber architecture. EverX Flow contains micrometer-scale glass fibers (140 µm, diameter 6 µm) with an aspect ratio (l/d) close to the range considered effective for reinforcement, which is between 30 and 94, while everX Posterior contains substantially longer, submillimeter-scale fibers (800 µm, diameter 17 µm) [67,68]. In contrast, the FibraFill composites are characterized by submillimeter-long fibers (800 µm) with a notably small, submicron diameter (0.7 µm). Although this combination results in an extremely high aspect ratio (>1000), these fibers may behave differently from the larger-diameter fibers used in everX materials. Although this was primarily discussed in relation to mechanical performance [67], microstructural differences may also influence polymerization behavior. The small fiber diameter of the FibraFill materials may have resulted in a substantially increased specific fiber surface area and possible fiber aggregation, which could potentially restrict monomer mobility and alter light propagation within the resin matrix, thereby reducing polymerization efficiency [69]. This might be one possible explanation of the significantly lower DC observed in the FibraFill materials compared with the everX materials in the present study. However, these mechanisms were not directly investigated in the present study and future studies combining polymerization analyses with detailed characterization of fiber architecture are required to clarify the potential influence on polymerization behavior.

A limitation of this study is that DC was evaluated only within the first 5 min after the start of polymerization, although it is established that polymerization of photo-activated composites continues after irradiation, leading to a gradual increase in DC over time [70,71]. However, as these delayed effects do not affect the relative ranking of materials [52], the results remain valid for comparison. Future studies could extend the observation period to more comprehensively assess the long-term development of DC. Real-time ATR-FTIR spectroscopy used in the present study is a well-established method for evaluating polymerization behavior in resin-based dental materials [64]. While this methodology enabled continuous monitoring of polymerization at the bottom surface of the specimens, future studies could complement these measurements with FTIR mapping to evaluate the spatial distribution of DC throughout the material.

## 5. Conclusions

The 3 s high-intensity photo-activation increased the polymerization rate but simultaneously reduced the degree of conversion of all investigated composites compared with a conventional 10 s light-curing regimen. The composites specifically designed for rapid high-intensity photo-activation exhibited similar or higher degree-of-conversion values than the investigated fiber-reinforced composites. The findings further suggest that the short-fiber-reinforced composites are more sensitive to high-intensity light-curing, while differences in fiber architecture may have contributed to the observed polymerization behavior.

## Figures and Tables

**Table 1:** Manufacturers’ information about the resin composite materials used in the study.

Composite Type	Composite Name	Composite Viscosity	Resin Matrix	Fiber	Photoinitiator	Filler Content (wt%/vol%)	Manufacturer	Shade/LOT No.
Conventional	Tetric PowerFill	High-viscosity	Bis-GMA, Bis-EMA, UDMA, Bis-PMA, DCP	-	CQ/amine +Ivocerin	77/54	Ivoclar, Schaan, Liechtenstein	IVA/Z09FBX
Conventional	Tetric PowerFlow	Flowable	Bis-GMA, Bis-EMA, UDMA	-	CQ/amine +Ivocerin	68/46	Ivoclar, Schaan, Liechtenstein	IVA/Z09HRG
SFRC	everX Posterior	High-viscosity	Bis-GMA, TEGDMA, PMMA	Short glass fiber (800 µm and Ø 17 µm)	CQ/amine	77/57	GC Corporation, Tokyo, Japan	A2/2502181
SFRC	everX Flow	Flowable	Bis-EMA, TEGDMA, UDMA	Short glass fiber (140 µm and Ø 6 µm)	CQ/amine	70/52	GC Corporation, Tokyo, Japan	Dentin shade/2504081
SFRC	FibraFill DENTIN	High-viscosity	EBPADMA, UDMA, TEGDMA	Short inorganic fibers (800 µm, Ø 0.7 µm)	CQ/amine	<80/–	ADM, Brno, Czech Republic	Bulk-fill shade/D_06-032025
SFRC	FibraFill Flow	Flowable	EBPADMA, UDMA, TEGDMA	Short inorganic fibers (800 µm, Ø 0.7 µm)	CQ/amine	<60/–	ADM, Brno, Czech Republic	Bulk-fill shade/F_05-112024

Bis-GMA: bisphenol-A-glycidyldimethacrylate, Bis-EMA: ethoxylated bisphenol-A-dimethacrylate, Bis-PMA: propoxylated bisphenol-A-dimethacrylate, TEGDMA: triethylene glycol dimethacrylate, UDMA: urethane dimethacrylate, DCP: tricyclodecan-dimethanol dimethacrylate, EBPADMA: ethoxylated bisphenol-A-dimethacrylate, CQ: camphorquinone, Ivocerin: bis-(4-methoxybenzoyl)diethylgermane, SFRC: short-fiber-reinforced composite.

## Data Availability

The original contributions presented in this study are included in the article. Further inquiries can be directed to the corresponding author.
